# Early mobilisation after abdominal surgery: a concept analysis

**DOI:** 10.1136/bmjopen-2025-107830

**Published:** 2026-04-17

**Authors:** Anna Schandl, Katinka Siesage, Anna-Karin Kroksmark, Ewa Gruber-De Sousa, Johanna Lilliecrona, Monika Fagevik Olsén

**Affiliations:** 1 Department of Perioperative and Intensive Care, Södersjukhuset, Stockholm, Sweden; 2 Department of Clinical Sciences and Education, Karolinska Institutet, Södersjukhuset, Stockholm, Sweden; 3 Department of Neuroscience and Physiology/Health and Rehabilitation, University of Gothenburg, Gothenburg, Sweden; 4 Department of Physiotherapy, Sahlgrenska University Hospital, Gothenburg, Sweden; 5 Department of Occupational Therapy and Physical Therapy, University of Gothenburg, Gothenburg, Sweden; 6 Department of Molecular and Clinical Medicine, Institute of Medicine, Sahlgrenska Academy, Gothenburg, Sweden

**Keywords:** Rehabilitation medicine, Adult surgery, Nursing Care, Hospitals, Physical Therapy Modalities

## Abstract

**Abstract:**

**Objective:**

To clarify and define the clinical practice concept of early mobilisation after abdominal surgery.

**Design:**

A concept analysis guided by Walker and Avant’s method.

**Data sources:**

MEDLINE (Ovid), AMED-(Ovid), Embase (Elsevier) and CINAHL (EBSCO) were searched through 5 December 2024.

**Eligibility criteria:**

Relevant studies that included combinations of the terms ‘early mobilisation’, ‘early ambulation’, ‘early acceleration’, ‘abdominal surgery’ and ‘surgical procedures’ were selected. We restricted the search to English full-text publications involving adult patients, limited to the year 2000 and onward. Inclusion criteria were original research articles describing the timing and/or type of mobilisation.

**Data extraction and synthesis:**

The study derives its defining attributes, antecedents and consequences through data analysis. To enhance understanding of the model, we constructed related and contrary cases of the concept and outlined relevant empirical referents.

**Results:**

In total, 140 studies were included in the analysis. Early mobilisation is characterised by the key defining attributes of initiating active physical movement, including standing, sitting in a chair or walking, within the first 24 hours of surgery. Antecedents include haemodynamic and respiratory stability, adequate pain management, and the patient’s cognitive and physical readiness. Contextual antecedents include competent and adequately staffed healthcare teams. Consequences include improved physiological recovery and enhanced postoperative outcomes.

**Conclusions:**

This analysis provides a clarified, practice-focused definition of early mobilisation after abdominal surgery. By delineating its key attributes and contextual prerequisites, the study offers a conceptual foundation that can support clinical guidelines, promote consistent implementation and inform future research aimed at optimising postoperative recovery.

**STRENGTHS AND LIMITATIONS OF THIS STUDY**

- The study applies a concept analysis method (Walker and Avant) to bring clarity to a clinically relevant practice concept.
- The analysis is based on a comprehensive search across four major databases.
- The findings depend on the quality, scope and conceptual clarity of available literature, which varied across included studies.
- Restricting the concept analysis to published literature may overlook tacit clinical knowledge and context-specific practices not captured in research.
- The method relies on the researcher’s interpretive judgement when identifying attributes, antecedents and consequences, which may introduce subjectivity despite systematic procedures.

## Background

 Early mobilisation is essential for postoperative recovery after abdominal surgery.^1^,^3^ Its primary purpose is to mitigate the adverse effects of prolonged bed rest, including deep vein thrombosis, pulmonary complications and muscle atrophy.^4^,^6^ Despite its recognised importance, there is limited knowledge regarding the timing and types of mobilisation that should be performed after specific surgeries.

Historically, mobilisation after surgery was considered harmful in the early 20th century.^7^ Over time, however, accumulating physiological and clinical evidence has demonstrated that immobilisation may contribute to cardiorespiratory complications and delayed postoperative recovery.^8^ Modern perioperative care guidelines, such as the Enhanced Recovery After Surgery (ERAS) pathway, recommend initiating physical activity shortly after surgery, even though few randomised, controlled trials have evaluated this practice.^9^,^12^ A major challenge in clinical practice and research is the absence of a universal definition of early mobilisation. This challenge is further complicated by variation in surgical techniques, anaesthesia methods, patient characteristics and clinical contexts.^13^ Abdominal surgery differs from many other surgical types because it elicits distinct physiological stress responses,^14^ carries specific postoperative risks and requires tailored rehabilitation strategies.^12^ Patients may face cardiorespiratory limitations^15^ and delays in mobilisation due to pain,^16^ incision location, impaired gastrointestinal function or reduced ability to generate effective ventilation and coughing^3 17 18^—particularly in procedures near the diaphragm.

Without a standard definition, decisions regarding the timing and type of mobilisation after abdominal surgery may vary between clinicians and institutions. This may lead to suboptimal recovery practices, reduced comparability of outcomes and challenges in implementing evidence-based physiotherapy interventions.^19^ Developing a clear definition improves comprehension of how early mobilisation is used, the factors influencing its implementation and its potential clinical impact. A holistic understanding of early mobilisation is important for advancing postoperative care and rehabilitation, especially within physiotherapy. Therefore, based on current literature, the study aims to clarify and define the clinical practice concept of early mobilisation after abdominal surgery.

## Materials and Methods

This study employed Walker and Avant’s method for concept analysis,^20^ which provides a structured process for identifying the defining attributes, antecedents and consequences of a clinical concept. Although early mobilisation after abdominal surgery includes physiological, experiential and organisational dimensions, the concept is fundamentally understood as a single clinical practice: the initiation of physical movement in the early postoperative period. Establishing conceptual boundaries around this practice ensures that the analysis remains coherent and appropriately aligned with Walker and Avant’s requirement for a clearly delimited concept.^20^

The decision to use Walker and Avant’s approach was based on its relevance for clarifying clinical concepts that lack definitional consistency yet play a central role in practice. This method has been widely applied in nursing and rehabilitation science to clarify similarly multifaceted but practice-focused phenomena, demonstrating its suitability for the present analysis.^20^ To ensure conceptual coherence, organisational, physiological and contextual factors influencing mobilisation are treated as antecedents or contextual prerequisites rather than as independent or overlapping concepts. The study is reported according to the guidelines of ‘The Preferred Reporting Items for Systematic reviews and Meta-Analyses (PRISMA) statement’ (online supplemental additional file: PRISMA 2020: checklist and PRISMA 2020 abstract checklist).^21^

### Literature search

**Figure 1:**
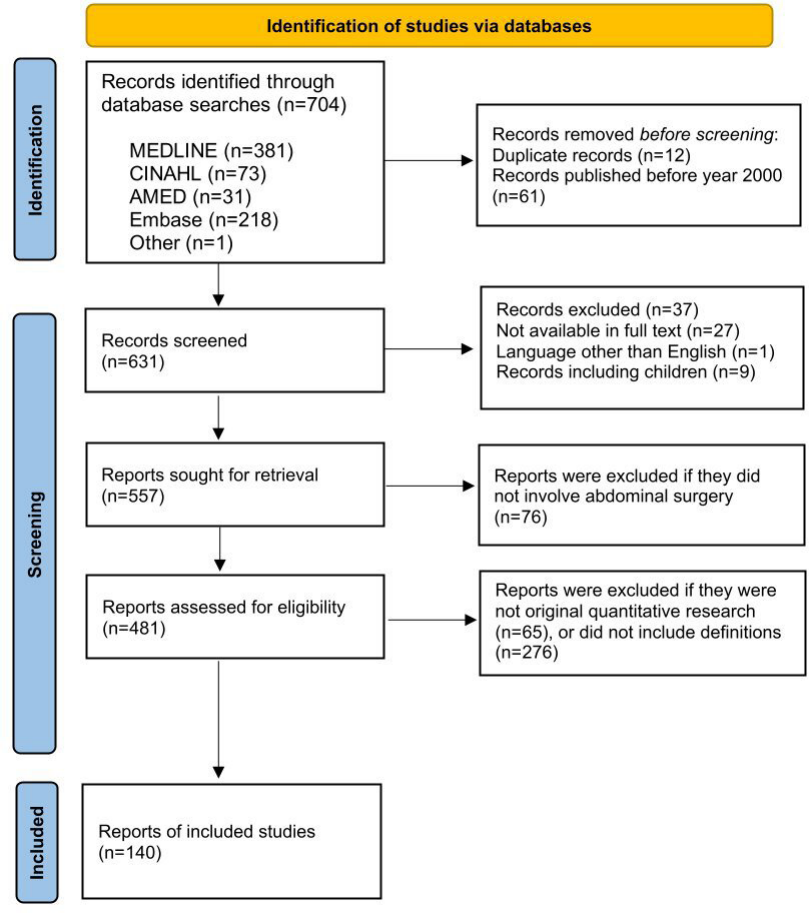
Flow chart demonstrating the selection process for the included articles.

The databases MEDLINE (Ovid), AMED (Ovid), Embase (Elsevier) and CINAHL (EBSCO) were searched for English language papers published that contain the following search terms: ‘*early mobilisation’, ‘early ambulation’, ‘early acceleration’, ‘abdominal surgery’, ‘surgical procedures’* (combined with types of abdominal surgeries), with synonyms or free text terms used separately or in combination. The full search strings and corresponding results are presented in the online supplemental file 1. Full search strings. The articles were retrieved through 5 December 2024. The reference lists of the included papers were searched to identify further sources. Inclusion criteria were original research articles in English, including adult subjects describing either timing and/or type of mobilisation. The literature search was restricted to studies published from 2000 onwards because ERAS programmes and modern evidence-based postoperative care pathways, within which early mobilisation is a core component, were introduced and widely implemented from the early 2000s.^22^ Earlier literature reflects different perioperative practices, surgical techniques and mobilisation protocols and was therefore unlikely to provide conceptually relevant or clinically comparable data for a contemporary analysis. The selection process for the included articles is illustrated in figure 1. The complete list of references can be found in online supplemental file 2. Dictionaries and textbooks were also searched for a definition of the concept separately (early AND mobilisation) and in combination (early mobilisation).

### The analysis process for deriving attributes

According to the method described by Walker and Avant, the following steps are included in the analysis: selecting a concept, determining the purpose of the analysis, identifying all uses of the concept, determining the defining attributes, identifying model, related and contrary cases, identifying antecedents and consequences and finally identifying the empirical referent.^20^

A total of 140 articles that met the inclusion criteria were reviewed in detail. From each study, we extracted statements, operational definitions and descriptions related to mobilisation activities and their timing after surgery. These data were then subjected to an iterative process of comparison and categorisation. First, all descriptions of mobilisation were categorised according to the type of activity described (eg, ambulation, standing, sitting out of bed). Second, timing-related descriptions were categorised according to when mobilisation was initiated. Third, the level of patient participation (active vs passive) was identified and categorised. During this iterative comparison, clusters of similar meanings emerged across studies. These clusters were then synthesised into preliminary conceptual categories. Each category was evaluated for (a) its recurrence across multiple studies, (b) centrality to the phenomenon of early mobilisation and (c) alignment with existing theoretical and clinical descriptions of postoperative mobilisation. Only characteristics that consistently appeared across the literature and were deemed essential to distinguishing early mobilisation from related but conceptually different phenomena (eg, passive transfer, routine postoperative activity) were retained as defining attributes.

### Patient involvement

None. The patients described in the model cases are fictitious and were created solely for illustrative purposes.

## Results

### Dictionary and thesaurus definitions

The Oxford English Dictionary defines ‘early’ as ‘before the proper or expected point in time’.^23^ According to the Wordsmyth English Dictionary–Thesaurus,^24^ the term ‘early’ means: in the first stage of a time; ahead of or before the usual or expected time.^24^ Synonyms of ‘early’ are ahead, before, beforehand, betimes and in advance.^24^ For mobilisation, the following definitions are found: To prepare for war of active service^23 25^; the act of assembling, organising or adapting for immediate use or service in times of war or emergency; the state of being mobilised.^24^ However, in the Swedish National Encyclopaedia, mobilisation is described as ‘a method within medical science enabling pain relief and regaining mobility function using manual hand manipulation.^26^ According to the medical thesaurus and the Swedish Academy Dictionary, mobilisation is defined as ‘making mobile’, which is the opposite of immobilisation, described as ‘immobility’.^25^

### Use of the concept/historical perspective

Already in 1899, the surgeon Emil Ries reported in an article that women undergoing vaginal abdominal procedures could safely be ambulated shortly after the surgery.^27^ He concluded that ambulation was feasible and associated with fewer wound complications and a shorter time until bowel normality. He started a paradigm shift from long periods of immobilisation towards more active postoperative movements. Since then, he has been considered to be the father of early postoperative ambulation.^7^ However, Ries was not the only one interested in changing the clinical practice of postoperative immobilisation. Another surgeon, Bolt, reported positive outcomes of early postoperative mobilisation.^28^ By 1907, these two surgeons had performed nearly 900 operations. In contrast to the long periods of postoperative immobility, which were common then, their patients were allowed to be out of bed and walk in the first postoperative days.^7 27 28^ Regardless of these reports, the paradigm shift to allow the implementation of early ambulation took time. Not until the mid-20th century did the change in clinical practice become more widespread, but some resistance remained.^29^ New trials showing fewer complications and faster recovery after minor surgery were published.^729^,^32^ Palumbo *et al*^31^ described the positive effects of ambulation (sitting on the edge of the bed or in a chair and walking in combination with deep breathing and leg exercises) within 24 hours after surgery.^31^ Early ambulation was associated with fewer postoperative complications, with no adverse events such as wound disruption, delayed wound healing or hernias. The ambulatory patients reported being more comfortable, having less pain and bronchial secretions, being more cooperative in the breathing exercises and developing fewer pulmonary complications. Despite some publications showing the beneficial effects of ambulation during this time,^729^,^32^ the level of evidence was low, since the studies mostly consist of case series or cohorts, statistical analyses are missing, and the results are often incompletely reported. In addition, the regimens of ambulation differed widely. Early postoperative ambulation was then and is still not clearly defined. In 1958, Powers suggested that prompt ambulation occurred on the day of surgery, early ambulation on the first day after surgery, late ambulation after the 10th postoperative day, and delayed ambulation was considered to occur between postoperative days 2 and 10.^7^ However, since these early studies were conducted, surgical procedures, anaesthesia, analgesia and preoperative and postoperative care have developed considerably. This makes it important to undertake new trials. During the last decades, several studies on early ambulation have been published, where ambulation has been included as a part of a postoperative intervention, such as in the ERAS programme.^2 33^ The ERAS society’s grade of recommendation for early ambulation is strong, despite the solitary effect of ambulation not yet being evaluated.^5^

### Derivation of the defining attributes

According to the 140 studies included, mobilisation was described in all of them and was most frequently defined as activities involving being out of bed. Specifically, mobilisation included ambulation/walking (n=54), sitting out of bed (n=24), standing (n=17), taking steps (n=8) and more generally being out of bed (n=39). In contrast, activities performed in bed (n=24) were less frequently associated with early mobilisation and more often linked to preparatory or passive exercises. This pattern established out-of-bed ambulation as an essential component.

The timing of initiation varied considerably across studies. Among the 140 studies that used the term ‘early mobilisation’ (ie, what was considered early) was defined in 135 studies, these definitions showed considerable variation. Mobilisation was described as beginning within 2 hours (n=5), within 4 hours (n=7), between 5–8 hours (n=11), within the first 24 hours (n=104) or within 48 hours (n=8) after arrival in the postoperative recovery unit. Given that the vast majority of studies (n=127) defined early mobilisation as beginning within the first 24 hours, this timeframe was identified as the attribute most consistently aligned with the concept.

Finally, through literature, mobilisation was repeatedly conceptualised as an active process requiring the patient’s engagement (eg, standing, transferring, taking steps or walking) rather than as a passive transfer performed entirely by staff. This distinction emerged as essential to differentiating early mobilisation from passive repositioning or dependency-related care interventions. Through this structured and iterative analytic process, three defining attributes of early mobilisation were identified:

1. Out-of-bed ambulation, including standing, taking steps, walking or sitting in a chair.
2. Initiation within the first 24 hours after surgery, consistent with the predominant definition of ‘early’ in the literature.
3. Active participation by the patient, distinguishing early mobilisation from passive movement or assisted transfers without patient effort.

These attributes align with theoretical understandings of early ambulation and support its documented physiological benefits, such as reducing postoperative atelectasis and pneumonia.^34 35^

#### Model case

According to Walker and Avant, a model case demonstrates all of the defined attributes of the concept.^20^ A model case for early mobilisation is described below:

A 61-year-old man underwent an elective colon resection for a tumour. The procedure was uneventful, and he awoke in the postoperative recovery unit shortly after surgery. He was tired and slightly dizzy but reported minimal pain and no nausea. His vital signs were stable.

A few hours later, he was transferred to the surgical ward, where the interprofessional team, consisting of a ward nurse, an assistant nurse and a physiotherapist, jointly assessed his postoperative status. During the afternoon ward round, the surgeon confirmed that the patient was medically stable for early mobilisation. The nurse reviewed his fluid balance, pain control and orthostatic vitals, while the physiotherapist evaluated his strength, balance and readiness to begin mobilisation. Based on this information, the team agreed to initiate early mobilisation the same afternoon. With assistance from both a nurse and a physiotherapist, the patient was helped to sit on the edge of the bed. He reported mild dizziness; the nurse monitored his blood pressure and oxygen saturation, which remained within acceptable ranges. As his symptoms stabilised, the physiotherapist guided him into standing with support. He tolerated the position well and walked a few steps to an armchair, where he sat for 10 min. He then became fatigued and mildly nauseous, and the nurse assisted him back to bed.

The next morning, the nurse encouraged him to sit on the bed edge independently, and his vital signs remained stable. In the afternoon, with the physiotherapist nearby to ensure safe mobilisation, he walked to the bathroom with minimal assistance. On postoperative day two, the interprofessional team reassessed him and agreed that he had regained sufficient strength and functional independence to be discharged home.

This case illustrates all defining attributes of early mobilisation. Mobilisation began on the day of surgery following a coordinated interdisciplinary assessment. The patient participated actively, receiving appropriate support calibrated to his needs. He temporarily experienced dizziness, fatigue and nausea but regained strength and functional capacity by the following day, enabling continued mobilisation with decreasing assistance and eventual independent activity. The collaboration between the medical doctor, nurse, assistant nurse and physiotherapist ensured timely and safe mobilisation.

#### Related case

Related cases are similar to the concept and relate to it in some way, but do not contain all the defining attributes, as shown in the following example:

A 75-year-old woman underwent an oesophagectomy for an oesophageal tumour. The surgery was lengthy and complex, and she returned to the recovery unit around midnight. After a few hours, she was awake but exhausted, intermittently disoriented and requiring high-flow oxygen due to her underlying chronic obstructive pulmonary disease and a history of recurrent pneumonia. The surgeon emphasised the importance of mobilisation to reduce pulmonary complications, but during the interprofessional morning assessment, the nurse and physiotherapist noted that she lacked the physical strength and cognitive clarity to participate actively. Despite these limitations, the team attempted a cautious effort to begin mobilisation. With two staff members supporting her, she was slowly transferred to a sitting position at the bedside. She remained passive throughout, unable to assist with balance or weight shifting. After a brief attempt to lift her into an armchair, she became pale and increasingly confused. Her blood pressure dropped, and the nurse immediately monitored her vital signs and recommended that she be returned to bed. The physiotherapist agreed that further mobilisation should be postponed until her condition stabilised and she could participate safely.

Although mobilisation was attempted, this case lacks key defining attributes of early mobilisation. The patient was unable to participate actively; her physiological instability prevented safe progression, and the interprofessional assessment did not support continued mobilisation. Thus, while the situation is similar in context, through the team’s initial intention to mobilise the patient, it does not represent a true example of the concept, which makes it a related case.

#### Contrary case

Contrary cases are examples of what the concept is not, for example:

A 70-year-old man underwent elective gastric bypass surgery. The procedure became complicated due to an unexpected intraoperative bleeding event. Although the bleeding was controlled, he remained haemodynamically unstable postoperatively. By the following day, most vital parameters had stabilised, except for intermittent episodes of atrial fibrillation that were being medically managed. His overall condition allowed for cautious mobilisation.

The nurse responsible for him was a newly graduated nurse and focused primarily on managing cardiac medications, intravenous infusions and continuous monitoring. It was a Saturday, so no physiotherapist was available, and the on-call physician conducted a brief round without addressing mobilisation. Feeling uncertain and lacking professional support from more experienced colleagues, the nurse did not initiate mobilisation. As a result, despite being clinically stable enough to start early activity, the patient remained in bed for the entire day.

This case represents the opposite of early mobilisation. Although the patient’s condition permitted activity, mobilisation did not occur due to organisational barriers, lack of interprofessional collaboration and insufficient staff competence. None of the defining attributes of early mobilisation, timely initiation, coordinated teamwork and patient engagement were present. Therefore, this is a contrary case, illustrating what early mobilisation is not.

### Antecedents and consequences

In this context, the antecedents include patients who are haemodynamically and respiratory stable, pain-relieved and cognitively and physically prepared to engage in mobilisation out of bed. Studies also highlight adherence to mobilisation protocols^36^,^38^ and the presence of knowledgeable and adequately staffed healthcare teams as prerequisites for early mobilisation.^38^,^40^ The intended consequence of early mobilisation is to prevent postoperative complications and, thus, accelerate recovery and return to preoperative physical status and independence/dependence in the activity of daily living. It may be assumed that patients who feel safe during their first mobilisation may be more likely to mobilise independently thereafter.^40^

### Empirical referents

Empirical referents are categories of phenomena that, by their existence, demonstrate the presence of the concept itself.^20^ The term ‘early’ refers to the time from arrival to postoperative care after surgery until a defined goal of mobilisation is achieved, is observable and can be measured in minutes or hours. The degree of mobilisation is visible and can be observed and registered. Mobilisation referents may include instruments evaluating physical functional outcomes. Wearable devices measuring mobilisation provide information based on the position and acceleration of the body and assess time spent sitting, lying, standing, stepping and the number of steps. Such devices have been used to measure the degree and time of mobility in patients who underwent abdominal surgery.^41^ Another empirical referent is the Activity Classification Guide for Inpatient Activities from the American College of Sports Medicine,^42^ a 6-point score describing activity classes of patients from sitting up in bed with assistance to independent ambulation, which has been used after cardiac surgery.^43^ However, none of these instruments include the time aspect preceding mobilisation.

## Discussion

The scientific literature commonly refers to early mobilisation, but a joint theoretical definition remains unrevealed. However, this study analysed the concept in the context of abdominal surgery. Based on current literature, the following definition, produced from this concept analysis, is: Early mobilisation is ambulation including out-of-bed activities such as sitting in a chair, standing and walking occurring within the first 24 hours of surgery, focusing on active rather than passive movement.

This definition underscores the dynamic nature of early mobilisation and its focus on active rather than passive movement. According to Walker and Avant, a concept analysis should not be regarded as complete but should reflect the critical elements of the concept during the current time.^20^ We believe that the present aspects of early mobilisation are captured in our definition. It must also be remembered that only articles published in English were reviewed, which may entail a certain degree of selection bias. This concept analysis is the first approach in a theory-building process for early mobilisation, but needs to be further explored across other surgeries and deeper theory synthesis. It must be kept in mind that this is a theoretical approach to early mobilisation. In clinical practice, several interrelated determinants influence both the timing and feasibility of early mobilisation, and decisions should always be based on the patient’s individual abilities and readiness. Important patient-related considerations include pre-existing comorbidities (eg, cardiopulmonary disease, frailty), postoperative physiological stability, effectiveness of pain management and the patient′s level of alertness.^44^ Anaesthesia techniques also play a significant role, as different methods can variably impact pain levels, respiratory function, motor block and overall readiness to engage in mobilisation.^45^ Beyond patient characteristics, variability in surgical procedures substantially affects the timing of mobilisation. For example, early mobilisation is often more achievable after minimally invasive abdominal procedures than after major surgeries that may involve greater postoperative respiratory compromise, closer haemodynamic monitoring or the presence of drains and lines that restrict movement.^46 47^ In addition, the attitudes, knowledge and clinical practices of healthcare professionals influence how consistently early mobilisation is implemented.^39^ Taken together, these differences across patient, surgical and organisational contexts underscore the challenge of establishing a unified definition of early mobilisation.

Despite these variations, the definition proposed in this concept analysis may support healthcare professionals in clinical practice by clarifying the essential attributes of early mobilisation. It may assist clinicians in assessing patient readiness, planning mobilisation goals and allocating physiotherapy resources efficiently. Furthermore, it may promote collaboration and joint understanding between nurses, physiotherapists and surgeons when planning and implementing postoperative mobilisation interventions.^48^

For research purposes, a common definition of early mobilisation may facilitate the evaluation of physiotherapy interventions. Most patients are willing to participate in early mobilisation after abdominal surgery and rely on healthcare professionals’ judgement of safe timing, duration and type of mobilisation.^40 41^ However, to determine the procedure’s safety, the first step is to establish a consensus on the definition. Hashem *et al*^49^ emphasised the significance of early mobilisation in the intensive care unit setting, extending its application beyond surgical patients to a wider range of critically ill individuals.^49^ This adaptability reflects the concept’s relevance across diverse clinical scenarios.

### Limitations

Walker and Avant’s concept analysis model is widely used in nursing and health sciences, but is not without limitations. The model follows a linear and static process, which may not fully capture the evolving nature of the concept. It also relies on existing literature, which can limit the depth of analysis if the literature is sparse or inconsistent. Alternative methodologies such as conceptual blending, hybrid concept development or theory synthesis could also be used to explore the wider conceptual ecosystem surrounding postoperative recovery practices. However, the primary purpose of this study was not to generate a new theory but to clarify a specific practice-related concept for clinical use. Thus, Walker and Avant’s structured approach was considered most appropriate for producing a clinically applicable definition.

In this study, although the available literature was extensive, some relevant studies not published in English may have been inadvertently excluded. Another limitation is the subjectivity involved in selecting defining attributes and illustrative cases, which can affect the consistency and replicability of the analysis. To mitigate this risk, researchers with diverse professional backgrounds and experiences were involved in the process. Nevertheless, the use of constructed case examples may oversimplify the complexity of real-world phenomena. Finally, the model lacks contextual and cultural sensitivity, which may limit its applicability across different settings. This underscores the importance of critically considering the concept’s relevance in its intended context.

## Conclusions

Early mobilisation after abdominal surgery encompasses a range of activities, predominantly focused on initiating ambulation and physical activity within the early postoperative period. While specific definitions may vary among studies and clinical contexts, the overarching goal remains consistent: to mitigate the adverse effects of immobility and expedite the patient’s recovery process. In this study, key defining attributes of early mobilisation include ambulation, such as standing, walking or sitting in a chair within the first 24 hours of surgery, focusing on active rather than passive movement. However, early mobilisation is not a one-size-fits-all approach but a dynamic and patient-centred strategy that healthcare providers tailor to individual surgical procedures, patient needs and clinical settings. Its profound impact on postoperative outcomes and recovery underscores the need for further research and development of standardised guidelines to optimise implementation.

## Supplementary material

10.1136/bmjopen-2025-107830 online supplemental file 1

10.1136/bmjopen-2025-107830 online supplemental file 2

10.1136/bmjopen-2025-107830 online supplemental file 3

10.1136/bmjopen-2025-107830 online supplemental file 4

## Data Availability

All data relevant to the study are included in the article or uploaded as supplementary information.
