# Supplementary material for: Early mobilisation after abdominal surgery: a concept analysis
Source: BMJ Open. 2026 Apr 17;16(4):e107830. doi: 10.1136/bmjopen-2025-107830 (PMC13110540; doi:10.1136/bmjopen-2025-107830)
Supplement: online supplemental file 1 [file bmjopen-16-4-s001.docx]

| **MEDLINE 2024-12-04** | | |
| --- | --- | --- |
|  | **Key words** | **Results** |
| **Block 1** | Early mobilization |  |
|  | **MeSH** |  |
| 1 | "Early Ambulation"[MeSH Terms] | 3 427 |
|  | **Synonyms** |  |
| 2 | "early mobilization"[Title/Abstract] OR "Early Ambulation"[Title/Abstract] OR "accelerated ambulation"[Title/Abstract] | 5 547 |
| 3 | 1 OR 2 | 7 632 |
| **Block 2** | Surgery |  |
|  | **MeSH** |  |
| 4 | abdomen/surgery"[MeSH Terms] OR "uterus/surgery"[MeSH Terms] OR "kidney/surgery"[MeSH Terms] OR "fallopian tubes/surgery"[MeSH Terms] OR "prostate/surgery"[MeSH Terms] OR "intestines/surgery"[MeSH Terms] OR "Thoracic Surgery"[MeSH Terms] OR "Colorectal Surgery"[MeSH Terms] OR "urologic surgical procedures, male"[MeSH Terms] OR "Gynecologic Surgical Procedures"[MeSH Terms] OR "Digestive System Surgical Procedures"[MeSH Terms] | 694 473 |
| 5 | 3 AND 4 |  |
| **6 Final** | **5 AND Limits: 2000-, English** | **381** |
|  | Link to references | <https://www.ncbi.nlm.nih.gov/sites/myncbi/1LeG-kvcZfoAq/collections/64783356/public/> |

Definition of search terms used in the selected database.

MeSH= Term from the Medline controlled vocabulary, including terms found below this term in the MeSH hierarchy, [Title/Abstract]=search Title and abstract

| **Cinahl 2024-12-04** | | |
| --- | --- | --- |
|  | **Key words** | **Results** |
| **Block 1** | Early mobilization |  |
|  | **Cinahl subject headings** |  |
| 1 | (MH "Early Ambulation") | 1 789 |
|  | **Synonyms** |  |
| 2 | TI ( "early mobilization" OR "early ambulation" OR “accelerated ambulation ) OR AB ( "early mobilization" OR "early ambulation" OR “accelerated ambulation ) | 1 771 |
| 3 | 1 OR 2 | 2 961 |
| **Block 2** | Surgery |  |
|  | **Cinahl subject headings** |  |
| 4 | (MH "Abdomen+/SU") OR (MH "Uterus+/SU") OR (MH "Kidney+/SU") OR (MH "Fallopian Tubes/SU") OR (MH "Prostate/SU") OR (MH "Intestines+/SU") OR (MH "Thoracic Surgery+") OR (MH "Digestive System+/SU") | 920508 |
| 5 | 3 AND 4 | 207 |
| **6 Final** | **5 AND Limits: 2000-, Eng, Peer review, Excl medline** | **73** |

MH = Medical Heading

"…"= Exact phrase

TI = Term find in title

AB = Term find in abstract

| **AMED-** |
| --- |

|  | **Key words** | **Results** |
| --- | --- | --- |
| **Block 1** | Early mobilization |  |
|  | **In text** |  |
| 1 | "early ambulation" OR "early mobilization" OR "early acceleration" OR "early ambulation" OR "early mobilization" OR "early acceleration" | 234 |
| **Block 2** | Surgery |  |
|  | **In text** |  |
| 2 | T surgery OR "surgical procedures" | 9 471 |
| 3 | 1 AND 2 | 54 |
| **4 Final** | **3 AND Limits: Eng, Peer review** | **31** |
|  |  |  |

| **Embase via Elsevier** | | |
| --- | --- | --- |
|  | **Key words** | **Results** |
| **Block 1** | Early mobilization |  |
|  | **Emtree** |  |
| 1 | 'mobilization'/exp | 43 338 |
|  | **Synonyms** |  |
| 2 | ((early AND mobilization OR early) AND ambulation OR accelerated) AND ambulation:ti,ab | 5 763 |
| 3 | 1 OR 2 | 45 575 |
| **Block 2** | Surgery |  |
|  | **Emtree** |  |
| 4 | 'abdomen'/exp/dm_su OR 'uterus'/exp/dm_su OR 'kidney'/exp/dm_su OR 'fallopian tube'/exp/dm_su OR 'prostate'/exp/dm_su OR 'intestine'/exp/dm_su OR 'thorax surgery'/exp OR 'colorectal surgery'/exp OR 'urologic surgery'/exp OR 'gynecologic surgery'/exp | 45 575 |
| 5 | 3 AND 4 | 4 611 |
| **6 Final** | **5 AND Limits: AND ('article'/it OR 'review'/it) AND [english]/lim AND ('article'/it OR 'review'/it) AND [english]/lim AND ('clinical trial'/de OR 'clinical trial topic'/de OR 'controlled study'/de OR 'meta analysis'/de OR 'randomized controlled trial'/de OR 'randomized controlled trial topic'/de OR 'systematic review'/de OR 'systematic review topic'/de)** | **218** |

/exp = Includes terms found below this term in the EMTREE hierarchy

/de = Term from the EMTREE controlled vocabulary

:ab,ti= Term found in title and/or abstract

:ab,ti,kw = Term found in title and/or abstract and/or keyword
