# Supplementary material for: Early mobilisation after abdominal surgery: a concept analysis
Source: BMJ Open. 2026 Apr 17;16(4):e107830. doi: 10.1136/bmjopen-2025-107830 (PMC13110540; doi:10.1136/bmjopen-2025-107830)
Supplement: online supplemental file 2 [file bmjopen-16-4-s002.docx]

Aggarwal, A., Irrinki, S., Kurdia, K. C., Khare, S., Naik, N., Tandup, C., Savlania, A., Dahiya, D., Kaman, L., & Sakaray, Y. (2023). Modified Enhanced Recovery After Surgery (ERAS) Protocol Versus Non-ERAS Protocol in Patients Undergoing Emergency Laparotomy for Acute Intestinal Obstruction: A Randomized Controlled Trial. *World J Surg*, *47*(12), 2990-2999. https://doi.org/10.1007/s00268-023-07176-1

Amari, T., Hirukawa, C., Fukuda, T., Hidaka, Y., Makita, Y., Fukuda, K., Sakamoto, Y., Kimura, M., & Yamaguchi, K. (2022). Factors Affecting the Length of Hospital Days After Laparoscopic Gastrectomy for Elderly Patients with Gastric Cancer. *J Gastrointest Cancer*, *53*(2), 472-479. https://doi.org/10.1007/s12029-021-00633-x

Archibald, L. H., Ott, M. J., Gale, C. M., Zhang, J., Peters, M. S., & Stroud, G. K. (2011). Enhanced recovery after colon surgery in a community hospital system. *Dis Colon Rectum*, *54*(7), 840-845. https://doi.org/10.1007/DCR.0b013e31821645bd

Asada, J., Ida, M., Sato, M., Okamoto, N., & Kawaguchi, M. (2019). Associated factors with delayed ambulation after abdominal surgery. *J Anesth*, *33*(6), 680-684. https://doi.org/10.1007/s00540-019-02696-4

Baird, G., Maxson, P., Wrobleski, D., & Luna, B. S. (2010). Fast-track colorectal surgery program reduces hospital length of stay. *Clin Nurse Spec*, *24*(4), 202-208. https://doi.org/10.1097/NUR.0b013e3181e3604c

Bakker, N., Cakir, H., Doodeman, H. J., & Houdijk, A. P. (2015). Eight years of experience with Enhanced Recovery After Surgery in patients with colon cancer: Impact of measures to improve adherence. *Surgery*, *157*(6), 1130-1136. https://doi.org/10.1016/j.surg.2015.01.016

Balvardi, S., Pecorelli, N., Castelino, T., Niculiseanu, P., Alhashemi, M., Liberman, A. S., Charlebois, P., Stein, B., Carli, F., Mayo, N. E., Feldman, L. S., & Fiore, J. F., Jr. (2021). Impact of Facilitation of Early Mobilization on Postoperative Pulmonary Outcomes After Colorectal Surgery: A Randomized Controlled Trial. *Ann Surg*, *273*(5), 868-875. https://doi.org/10.1097/sla.0000000000003919

Bansal, D., Nayak, B., Singh, P., Nayyar, R., Ramachandran, R., Kumar, R., & Seth, A. (2020). Randomized controlled trial to compare outcomes with and without the enhanced recovery after surgery protocol in patients undergoing radical cystectomy [Article]. *Indian Journal of Urology*, *36*(2), 95-100. https://doi.org/10.4103/iju.IJU_11_20

Basse, L., Thorbøl, J. E., Løssl, K., & Kehlet, H. (2004). Colonic surgery with accelerated rehabilitation or conventional care. *Dis Colon Rectum*, *47*(3), 271-277; discussion 277-278. https://doi.org/10.1007/s10350-003-0055-0

Bergman, S., Deban, M., Martelli, V., Monette, M., Sourial, N., Hamadani, F., Teasdale, D., Holcroft, C., Zakrzewski, H., & Fraser, S. (2014). Association between quality of care and complications after abdominal surgery. *Surgery*, *156*(3), 632-639. https://doi.org/10.1016/j.surg.2013.12.031

Blom, R. L., van Heijl, M., Bemelman, W. A., Hollmann, M. W., Klinkenbijl, J. H., Busch, O. R., & van Berge Henegouwen, M. I. (2013). Initial experiences of an enhanced recovery protocol in esophageal surgery. *World J Surg*, *37*(10), 2372-2378. https://doi.org/10.1007/s00268-013-2135-1

Boden, I., El-Ansary, D., Zalucki, N., Robertson, I. K., Browning, L., Skinner, E. H., & Denehy, L. (2018). Physiotherapy education and training prior to upper abdominal surgery is memorable and has high treatment fidelity: a nested mixed-methods randomised-controlled study. *Physiotherapy*, *104*(2), 194-202. https://doi.org/10.1016/j.physio.2017.08.008

Boden, I., Skinner, E. H., Browning, L., Reeve, J., Anderson, L., Hill, C., Robertson, I. K., Story, D., & Denehy, L. (2018). Preoperative physiotherapy for the prevention of respiratory complications after upper abdominal surgery: pragmatic, double blinded, multicentre randomised controlled trial. *Bmj*, *360*, j5916. https://doi.org/10.1136/bmj.j5916

Boden, I., Sullivan, K., Hackett, C., Winzer, B., Lane, R., McKinnon, M., & Robertson, I. (2018). ICEAGE (Incidence of Complications following Emergency Abdominal surgery: Get Exercising): study protocol of a pragmatic, multicentre, randomised controlled trial testing physiotherapy for the prevention of complications and improved physical recovery after emergency abdominal surgery. *World J Emerg Surg*, *13*, 29. https://doi.org/10.1186/s13017-018-0189-y

Boitano, T. K. L., Smith, H. J., Rushton, T., Johnston, M. C., Lawson, P., Leath, C. A., 3rd, Xhaja, A., Guthrie, M. P., & Straughn, J. M., Jr. (2018). Impact of enhanced recovery after surgery (ERAS) protocol on gastrointestinal function in gynecologic oncology patients undergoing laparotomy. *Gynecol Oncol*, *151*(2), 282-286. https://doi.org/10.1016/j.ygyno.2018.09.009

Braga, M., Pecorelli, N., Ariotti, R., Capretti, G., Greco, M., Balzano, G., Castoldi, R., & Beretta, L. (2014). Enhanced recovery after surgery pathway in patients undergoing pancreaticoduodenectomy. *World J Surg*, *38*(11), 2960-2966. https://doi.org/10.1007/s00268-014-2653-5

Braga, M., Pecorelli, N., Scatizzi, M., Borghi, F., Missana, G., & Radrizzani, D. (2017). Enhanced Recovery Program in High-Risk Patients Undergoing Colorectal Surgery: Results from the PeriOperative Italian Society Registry. *World J Surg*, *41*(3), 860-867. https://doi.org/10.1007/s00268-016-3766-9

Browning, L., Denehy, L., & Scholes, R. L. (2007). The quantity of early upright mobilisation performed following upper abdominal surgery is low: an observational study. *Aust J Physiother*, *53*(1), 47-52. https://doi.org/10.1016/s0004-9514(07)70061-2

Bundgaard-Nielsen, M., Jans, Ø., Müller, R. G., Korshin, A., Ruhnau, B., Bie, P., Secher, N. H., & Kehlet, H. (2013). Does goal-directed fluid therapy affect postoperative orthostatic intolerance?: A randomized trial. *Anesthesiology*, *119*(4), 813-823. https://doi.org/10.1097/ALN.0b013e31829ce4ea

Bundgaard-Nielsen, M., Jørgensen, C. C., Jørgensen, T. B., Ruhnau, B., Secher, N. H., & Kehlet, H. (2009). Orthostatic intolerance and the cardiovascular response to early postoperative mobilization. *Br J Anaesth*, *102*(6), 756-762. https://doi.org/10.1093/bja/aep083

Cerfolio, R. J., Bryant, A. S., Bass, C. S., Alexander, J. R., & Bartolucci, A. A. (2004). Fast tracking after Ivor Lewis esophagogastrectomy. *Chest*, *126*(4), 1187-1194. https://doi.org/10.1378/chest.126.4.1187

Chen, C. C., Chen, C. N., Lai, I. R., Huang, G. H., Saczynski, J. S., & Inouye, S. K. (2014). Effects of a modified Hospital Elder Life Program on frailty in individuals undergoing major elective abdominal surgery. *J Am Geriatr Soc*, *62*(2), 261-268. https://doi.org/10.1111/jgs.12651

Chen, C. C., Li, H. C., Liang, J. T., Lai, I. R., Purnomo, J. D. T., Yang, Y. T., Lin, B. R., Huang, J., Yang, C. Y., Tien, Y. W., Chen, C. N., Lin, M. T., Huang, G. H., & Inouye, S. K. (2017). Effect of a Modified Hospital Elder Life Program on Delirium and Length of Hospital Stay in Patients Undergoing Abdominal Surgery: A Cluster Randomized Clinical Trial. *JAMA Surg*, *152*(9), 827-834. https://doi.org/10.1001/jamasurg.2017.1083

Chen, C. C., Lin, M. T., Tien, Y. W., Yen, C. J., Huang, G. H., & Inouye, S. K. (2011). Modified hospital elder life program: effects on abdominal surgery patients. *J Am Coll Surg*, *213*(2), 245-252. https://doi.org/10.1016/j.jamcollsurg.2011.05.004

Daenen, C., Coimbra, C., Hans, G., & Joris, J. (2018). Labelling as reference Centre of GRACE (Groupe francophone de Réhabilitation Améliorée après ChirurgiE) for colorectal surgery: its impact on the implementation of enhanced recovery programme at the University Hospital of Liège. *Acta Chir Belg*, *118*(5), 294-298. https://doi.org/10.1080/00015458.2018.1427837

Day, R. W., Cleeland, C. S., Wang, X. S., Fielder, S., Calhoun, J., Conrad, C., Vauthey, J. N., Gottumukkala, V., & Aloia, T. A. (2015). Patient-Reported Outcomes Accurately Measure the Value of an Enhanced Recovery Program in Liver Surgery. *J Am Coll Surg*, *221*(6), 1023-1030.e1021-1022. https://doi.org/10.1016/j.jamcollsurg.2015.09.011

de Almeida, E. P. M., de Almeida, J. P., Landoni, G., Galas, F., Fukushima, J. T., Fominskiy, E., de Brito, C. M. M., Cavichio, L. B. L., de Almeida, L. A. A., Ribeiro, U., Jr., Osawa, E. A., Diz, M. P., Cecatto, R. B., Battistella, L. R., & Hajjar, L. A. (2017). Early mobilization programme improves functional capacity after major abdominal cancer surgery: a randomized controlled trial. *Br J Anaesth*, *119*(5), 900-907. https://doi.org/10.1093/bja/aex250

De Pasqual, C. A., Torroni, L., Gervasi, M. C., Alberti, L., Mengardo, V., Benedetti, B., Giacopuzzi, S., & Weindelmayer, J. (2020). Feasibility and safety of an enhanced recovery protocol (ERP) for upper GI surgery in elderly patients (≥ 75 years) in a high-volume surgical center. *Updates Surg*, *72*(3), 751-760. https://doi.org/10.1007/s13304-020-00824-4

Delaney, C. P., Zutshi, M., Senagore, A. J., Remzi, F. H., Hammel, J., & Fazio, V. W. (2003). Prospective, randomized, controlled trial between a pathway of controlled rehabilitation with early ambulation and diet and traditional postoperative care after laparotomy and intestinal resection. *Dis Colon Rectum*, *46*(7), 851-859. https://doi.org/10.1007/s10350-004-6672-4

Fagevik Olsén, M., Becovic, S., & Dean, E. (2021). Short-term effects of mobilization on oxygenation in patients after open surgery for pancreatic cancer: a randomized controlled trial. *BMC Surg*, *21*(1), 185. https://doi.org/10.1186/s12893-021-01187-2

Fischer, C. P., Knapp, L., Cohen, M. E., Ko, C. Y., Reinke, C. E., & Wick, E. C. (2021). Feasibility of Enhanced Recovery in Emergency Colorectal Operation. *J Am Coll Surg*, *232*(2), 178-185. https://doi.org/10.1016/j.jamcollsurg.2020.10.004

Fukushima, T., Adachi, T., Hanada, M., Tanaka, T., Oikawa, M., Nagura, H., Eguchi, S., & Kozu, R. (2021). Role of Early Mobilization on the Clinical Course of Patients who Underwent Pancreaticoduodenectomy: A Retrospective Cohort Study. *Tohoku J Exp Med*, *254*(4), 287-294. https://doi.org/10.1620/tjem.254.287

Gemma, M., Pennoni, F., & Braga, M. (2021). Studying Enhanced Recovery After Surgery (ERAS®) Core Items in Colorectal Surgery: A Causal Model with Latent Variables. *World J Surg*, *45*(4), 928-939. https://doi.org/10.1007/s00268-020-05940-1

Geubbels, N., Bruin, S. C., Acherman, Y. I., van de Laar, A. W., Hoen, M. B., & de Brauw, L. M. (2014). Fast track care for gastric bypass patients decreases length of stay without increasing complications in an unselected patient cohort. *Obes Surg*, *24*(3), 390-396. https://doi.org/10.1007/s11695-013-1133-6

Giacopuzzi, S., Weindelmayer, J., Treppiedi, E., Bencivenga, M., Ceola, M., Priolo, S., Carlini, M., & de Manzoni, G. (2017). Enhanced recovery after surgery protocol in patients undergoing esophagectomy for cancer: a single center experience. *Dis Esophagus*, *30*(4), 1-6. https://doi.org/10.1093/dote/dow024

Gillissen, F., Ament, S. M., Maessen, J. M., Dejong, C. H., Dirksen, C. D., van der Weijden, T., & von Meyenfeldt, M. F. (2015). Sustainability of an enhanced recovery after surgery program (ERAS) in colonic surgery. *World J Surg*, *39*(2), 526-533. https://doi.org/10.1007/s00268-014-2744-3

Gonzalez-Ayora, S., Pastor, C., Guadalajara, H., Ramirez, J. M., Royo, P., Redondo, E., Arroyo, A., Moya, P., & Garcia-Olmo, D. (2016). Enhanced recovery care after colorectal surgery in elderly patients. Compliance and outcomes of a multicenter study from the Spanish working group on ERAS. *Int J Colorectal Dis*, *31*(9), 1625-1631. https://doi.org/10.1007/s00384-016-2621-7

Grass, F., Pache, B., Martin, D., Addor, V., Hahnloser, D., Demartines, N., & Hübner, M. (2018). Feasibility of early postoperative mobilisation after colorectal surgery: A retrospective cohort study. *Int J Surg*, *56*, 161-166. https://doi.org/10.1016/j.ijsu.2018.06.024

Gustafsson, U. O., Oppelstrup, H., Thorell, A., Nygren, J., & Ljungqvist, O. (2016). Adherence to the ERAS protocol is Associated with 5-Year Survival After Colorectal Cancer Surgery: A Retrospective Cohort Study. *World J Surg*, *40*(7), 1741-1747. https://doi.org/10.1007/s00268-016-3460-y

Haines, K. J., Skinner, E. H., & Berney, S. (2013). Association of postoperative pulmonary complications with delayed mobilisation following major abdominal surgery: An observational cohort study [Journal Article]. *Physiotherapy (London)*, *99*(2), 119-125. https://sll.idm.oclc.org/login?url=https://search.ebscohost.com/login.aspx?direct=true&db=amed&AN=0167529&lang=sv&site=ehost-live

Hampton, J. P., Owodunni, O. P., Bettick, D., Chen, S. Y., Sateri, S., Magnuson, T., & Gearhart, S. L. (2019). Compliance to an enhanced recovery pathway among patients with a high frailty index after major gastrointestinal surgery results in improved 30-day outcomes. *Surgery*, *166*(1), 75-81. https://doi.org/10.1016/j.surg.2019.01.027

Hanada, M., Hidaka, M., Soyama, A., Tanaka, T., Hara, T., Matsushima, H., Haraguchi, M., Kitamura, M., Sekino, M., Oikawa, M., Nagura, H., Takeuchi, R., Sato, S., Takahata, H., Eguchi, S., & Kozu, R. (2022). Association between hospital acquired disability and post-discharge mortality in patients after living donor liver transplantation. *BMC Surg*, *22*(1), 445. https://doi.org/10.1186/s12893-022-01896-2

Hao, Y., Zhao, Q., Jiang, K., Feng, X., Ma, Y., Zhang, J., Han, X., Ji, G., Dong, H., & Nie, H. (2024). Association of adherence to the enhanced recovery after surgery pathway and outcomes after laparoscopic total gastrectomy. *BMC Anesthesiol*, *24*(1), 110. https://doi.org/10.1186/s12871-024-02433-9

Hattori, K., Mizuno, Y., Ogura, Y., Inoue, T., Nagaya, M., Jobara, K., Kurata, N., & Nishida, Y. (2022). Effects of Neuromuscular Electrical Stimulation on Lower Limb Muscle Strength After Living Donor Liver Transplant: A Case-Control Study. *Transplant Proc*, *54*(3), 749-754. https://doi.org/10.1016/j.transproceed.2022.01.028

Henriksen, M. G., Hessov, I., Dela, F., Hansen, H. V., Haraldsted, V., & Rodt, S. A. (2003). Effects of preoperative oral carbohydrates and peptides on postoperative endocrine response, mobilization, nutrition and muscle function in abdominal surgery. *Acta Anaesthesiol Scand*, *47*(2), 191-199. https://doi.org/10.1034/j.1399-6576.2003.00047.x

Henriksen, M. G., Jensen, M. B., Hansen, H. V., Jespersen, T. W., & Hessov, I. (2002). Enforced mobilization, early oral feeding, and balanced analgesia improve convalescence after colorectal surgery. *Nutrition*, *18*(2), 147-152. https://doi.org/10.1016/s0899-9007(01)00748-1

Hjort Jakobsen, D., Sonne, E., Basse, L., Bisgaard, T., & Kehlet, H. (2004). Convalescence after colonic resection with fast-track versus conventional care. *Scand J Surg*, *93*(1), 24-28. https://doi.org/10.1177/145749690409300105

Hussey, J. M., Yang, T., Dowds, J., O'Connor, L., Reynolds, J. V., & Guinan, E. M. (2019). Quantifying postoperative mobilisation following oesophagectomy [Journal Article]. *Physiotherapy (London)*, *105*(1), 126-133. https://sll.idm.oclc.org/login?url=https://search.ebscohost.com/login.aspx?direct=true&db=amed&AN=5006430&lang=sv&site=ehost-live

Ionescu, D., Iancu, C., Ion, D., Al-Hajjar, N., Margarit, S., Mocan, L., Mocan, T., Deac, D., Bodea, R., & Vasian, H. (2009). Implementing fast-track protocol for colorectal surgery: a prospective randomized clinical trial. *World J Surg*, *33*(11), 2433-2438. https://doi.org/10.1007/s00268-009-0197-x

Jian, C., Fang, J., Wu, L., Zheng, Z., Song, Y., Liu, W., Lin, X., & Yang, C. (2021). Failure of enhanced recovery programs after laparoscopic radical gastrectomy: a single-center retrospective study. *Surg Endosc*, *35*(6), 2629-2635. https://doi.org/10.1007/s00464-020-07683-5

Jones, B. A., Brock, B., Richman, J., Wood, L., Harsono, A. A. H., Oslock, W. M., English, N. C., Rubyan, M., & Chu, D. I. (2024). Which individual components of a colorectal surgery enhanced recovery program are associated with improved surgical outcomes? *Surgery*, *176*(4), 1044-1051. https://doi.org/10.1016/j.surg.2024.06.015

Jones, C., Kelliher, L., Dickinson, M., Riga, A., Worthington, T., Scott, M. J., Vandrevala, T., Fry, C. H., Karanjia, N., & Quiney, N. (2013). Randomized clinical trial on enhanced recovery versus standard care following open liver resection. *Br J Surg*, *100*(8), 1015-1024. https://doi.org/10.1002/bjs.9165

Jurt, J., Hübner, M., Pache, B., Hahnloser, D., Demartines, N., & Grass, F. (2018). Respiratory Complications After Colorectal Surgery: Avoidable or Fate? *World J Surg*, *42*(9), 2708-2714. https://doi.org/10.1007/s00268-018-4699-2

Jønsson, L. R., Foss, N. B., Orbæk, J., Lauritsen, M. L., Sejrsen, H. N., & Kristensen, M. T. (2023). Early intensive mobilization after acute high-risk abdominal surgery: a nonrandomized prospective feasibility trial. *Can J Surg*, *66*(3), E236-e245. https://doi.org/10.1503/cjs.008722

Jønsson, L. R., Ingelsrud, L. H., Tengberg, L. T., Bandholm, T., Foss, N. B., & Kristensen, M. T. (2018). Physical performance following acute high-risk abdominal surgery: a prospective cohort study. *Can J Surg*, *61*(1), 42-49. https://doi.org/10.1503/cjs.012616

Kapritsou, M., Korkolis, D. P., Giannakopoulou, M., Kaklamanos, I., Konstantinou, M., Katsoulas, T., Kiekkas, P., & Konstantinou, E. A. (2018). Fast-Track Recovery Program After Major Liver Resection: A Randomized Prospective Study. *Gastroenterol Nurs*, *41*(2), 104-110. https://doi.org/10.1097/sga.0000000000000306

Kasalar, I., & Sarigol Ordin, Y. (2024). Relation of Early Mobility With Gastrointestinal Symptoms and Pain in Patients Undergoing Abdominal Surgery. *J Perianesth Nurs*, *39*(4), 604-610. https://doi.org/10.1016/j.jopan.2023.11.001

Katogi, M. (2020). Comparison of life-behavior-promoting mobilization care with walking-only mobilization care in post-gastrointestinal surgery patients: A quasi-experimental study. *Jpn J Nurs Sci*, *17*(4), e12348. https://doi.org/10.1111/jjns.12348

Kim, J. W., Kim, W. S., Cheong, J. H., Hyung, W. J., Choi, S. H., & Noh, S. H. (2012). Safety and efficacy of fast-track surgery in laparoscopic distal gastrectomy for gastric cancer: a randomized clinical trial. *World J Surg*, *36*(12), 2879-2887. https://doi.org/10.1007/s00268-012-1741-7

Kırtıl, İ., Kanan, N., & Karip, A. B. (2023). Effects of a Mobilization Program Applied to Bariatric Surgery Patients on Preventing Gastrointestinal Complications: a Quasi-Experimental Study. *Obes Surg*, *33*(6), 1820-1830. https://doi.org/10.1007/s11695-023-06609-z

Kovar, A., Carmichael, H., Jones, T. S., Hosokawa, P., Goode, C. M., Overbey, D. M., Jones, E. L., & Robinson, T. N. (2022). Early identification of patients at risk for delayed recovery of ambulation after elective abdominal surgery. *Surg Endosc*, *36*(7), 4828-4833. https://doi.org/10.1007/s00464-021-08829-9

Kummer, A., Slieker, J., Grass, F., Hahnloser, D., Demartines, N., & Hübner, M. (2016). Enhanced Recovery Pathway for Right and Left Colectomy: Comparison of Functional Recovery. *World J Surg*, *40*(10), 2519-2527. https://doi.org/10.1007/s00268-016-3563-5

Lam, J., Suzuki, T., Bernstein, D., Zhao, B., Maeda, C., Pham, T., Sandler, B. J., Jacobsen, G. R., Cheverie, J. N., & Horgan, S. (2019). An ERAS protocol for bariatric surgery: is it safe to discharge on post-operative day 1? *Surg Endosc*, *33*(2), 580-586. https://doi.org/10.1007/s00464-018-6368-9

Lee, J. A., Young, S., O'Connor, V., & DiFronzo, L. A. (2020). Safety and Efficacy of an Enhanced Recovery Protocol After Hepatic Resection. *Am Surg*, *86*(10), 1396-1400. https://doi.org/10.1177/0003134820964492

Lee, S. S., Chern, J. Y., Frey, M. K., Comfort, A., Lee, J., Roselli, N., & Boyd, L. R. (2021). Enhanced recovery Pathways in gynecologic surgery: Are they safe and effective in the elderly? [Article]. *Gynecologic Oncology Reports*, *38*. https://doi.org/10.1016/j.gore.2021.100862

Lee, T. G., Kang, S. B., Kim, D. W., Hong, S., Heo, S. C., & Park, K. J. (2011). Comparison of early mobilization and diet rehabilitation program with conventional care after laparoscopic colon surgery: a prospective randomized controlled trial. *Dis Colon Rectum*, *54*(1), 21-28. https://doi.org/10.1007/DCR.0b013e3181fcdb3e

Levy, B. F., Scott, M. J., Fawcett, W. J., & Rockall, T. A. (2009). 23-hour-stay laparoscopic colectomy. *Dis Colon Rectum*, *52*(7), 1239-1243. https://doi.org/10.1007/DCR.0b013e3181a0b32d

Li, H., Luo, T. F., Zhang, N. R., Zhang, L. Z., Huang, X., & Jin, S. Q. (2022). Factors associated with prolonged postoperative length of hospital stay after laparoscopic colorectal cancer resection: a secondary analysis of a randomized controlled trial. *BMC Surg*, *22*(1), 438. https://doi.org/10.1186/s12893-022-01886-4

Li, Z., Zhou, L., Li, M., Wang, W., Wang, L., Dong, W., Chen, J., & Gong, S. (2024). Early mobilization after pancreatic surgery: A randomized controlled trial. *Surgery*, *176*(4), 1179-1188. https://doi.org/10.1016/j.surg.2024.06.027

Liang, X., Ying, H., Wang, H., Xu, H., Liu, M., Zhou, H., Ge, H., Jiang, W., Feng, L., Liu, H., Zhang, Y., Mao, Z., Li, J., Shen, B., Liang, Y., & Cai, X. (2018). Enhanced recovery care versus traditional care after laparoscopic liver resections: a randomized controlled trial. *Surg Endosc*, *32*(6), 2746-2757. https://doi.org/10.1007/s00464-017-5973-3

Lin, C., Wan, F., Lu, Y., Li, G., Yu, L., & Wang, M. (2019). Enhanced recovery after surgery protocol for prostate cancer patients undergoing laparoscopic radical prostatectomy. *J Int Med Res*, *47*(1), 114-121. https://doi.org/10.1177/0300060518796758

Lin, J. H., Whelan, R. L., Sakellarios, N. E., Cekic, V., Forde, K. A., Bank, J., & Feingold, D. L. (2009). Prospective study of ambulation after open and laparoscopic colorectal resection. *Surg Innov*, *16*(1), 16-20. https://doi.org/10.1177/1553350608330478

Lohsiriwat, V. (2019). Enhanced recovery after surgery for emergency colorectal surgery: Are there any differences between intra-abdominal infection and other indications? *J Visc Surg*, *156*(6), 489-496. https://doi.org/10.1016/j.jviscsurg.2019.05.006

Low, D. E., Kunz, S., Schembre, D., Otero, H., Malpass, T., Hsi, A., Song, G., Hinke, R., & Kozarek, R. A. (2007). Esophagectomy--it's not just about mortality anymore: standardized perioperative clinical pathways improve outcomes in patients with esophageal cancer. *J Gastrointest Surg*, *11*(11), 1395-1402; discussion 1402. https://doi.org/10.1007/s11605-007-0265-1

Ludwig, K., Enker, W. E., Delaney, C. P., Wolff, B. G., Du, W., Fort, J. G., Cherubini, M., Cucinotta, J., & Techner, L. (2008). Gastrointestinal tract recovery in patients undergoing bowel resection: results of a randomized trial of alvimopan and placebo with a standardized accelerated postoperative care pathway. *Arch Surg*, *143*(11), 1098-1105. https://doi.org/10.1001/archsurg.143.11.1098

Mackay, M. R., Ellis, E., & Johnston, C. (2005). Randomised clinical trial of physiotherapy after open abdominal surgery in high risk patients. *Aust J Physiother*, *51*(3), 151-159. https://doi.org/10.1016/s0004-9514(05)70021-0

Madan, S., Sureshkumar, S., Anandhi, A., Gurushankari, B., Keerthi, A. R., Palanivel, C., Kundra, P., & Kate, V. (2023). Comparison of Enhanced Recovery After Surgery (ERAS) Pathway Versus Standard Care in Patients Undergoing Elective Stoma Reversal Surgery- A Randomized Controlled Trial. *J Gastrointest Surg*, *27*(11), 2667-2675. https://doi.org/10.1007/s11605-023-05803-9

Mahendran, R., Tewari, M., Dixit, V. K., & Shukla, H. S. (2019). Enhanced recovery after surgery protocol enhances early postoperative recovery after pancreaticoduodenectomy. *Hepatobiliary Pancreat Dis Int*, *18*(2), 188-193. https://doi.org/10.1016/j.hbpd.2018.12.005

Miller, T. E., Thacker, J. K., White, W. D., Mantyh, C., Migaly, J., Jin, J., Roche, A. M., Eisenstein, E. L., Edwards, R., Anstrom, K. J., Moon, R. E., & Gan, T. J. (2014). Reduced length of hospital stay in colorectal surgery after implementation of an enhanced recovery protocol. *Anesth Analg*, *118*(5), 1052-1061. https://doi.org/10.1213/ane.0000000000000206

Minig, L., Chuang, L., Patrono, M. G., Fernandez-Chereguini, M., Cárdenas-Rebollo, J. M., & Biffi, R. (2015). Clinical outcomes after fast-track care in women undergoing laparoscopic hysterectomy. *Int J Gynaecol Obstet*, *131*(3), 301-304. https://doi.org/10.1016/j.ijgo.2015.06.034

Morais de Babo, N. M., Filipe Lima Barbosa, C., Almeida Ferreira, A. L., & Silva, L. I. (2023). ERAS programme in a Portuguese tertiary hospital: An audit of the first six months of implementation in elective colorectal surgery. *Rev Esp Anestesiol Reanim (Engl Ed)*, *70*(5), 247-258. https://doi.org/10.1016/j.redare.2022.04.001

Munk-Madsen, P., Eriksen, J. R., Kehlet, H., & Gögenur, I. (2019). Why still in hospital after laparoscopic colorectal surgery within an enhanced recovery programme? *Colorectal Dis*, *21*(12), 1438-1444. https://doi.org/10.1111/codi.14762

Nechay, T., Sazhin, A., Titkova, S., Tyagunov, A., Anurov, M., Melnikov-Makarchuk, K., & Tyagunov, A. (2020). Evaluation of enhanced recovery after surgery program components implemented in laparoscopic appendectomy: prospective randomized clinical study. *Sci Rep*, *10*(1), 10749. https://doi.org/10.1038/s41598-020-67591-5

Nevo, Y., Shaltiel, T., Constantini, N., Rosin, D., Gutman, M., Zmora, O., & Nevler, A. (2022). Activity Tracking After Surgery: Does It Correlate With Postoperative Complications? *Am Surg*, *88*(2), 226-232. https://doi.org/10.1177/0003134820988818

Ni, C. Y., Wang, Z. H., Huang, Z. P., Zhou, H., Fu, L. J., Cai, H., Huang, X. X., Yang, Y., Li, H. F., & Zhou, W. P. (2018). Early enforced mobilization after liver resection: A prospective randomized controlled trial. *Int J Surg*, *54*(Pt A), 254-258. https://doi.org/10.1016/j.ijsu.2018.04.060

Nishijima, M., Baba, H., Murotani, K., Tokai, R., Watanabe, T., Hirano, K., Shibuya, K., Hojo, S., Matsui, K., Yoshioka, I., Okumura, T., & Fujii, T. (2020). Early ambulation after general and digestive surgery: a retrospective single-center study. *Langenbecks Arch Surg*, *405*(5), 613-622. https://doi.org/10.1007/s00423-020-01925-9

Niwa, Y., Koike, M., Hattori, M., Iwata, N., Takami, H., Hayashi, M., Kanda, M., Kobayashi, D., Tanaka, C., Yamada, S., Fujii, T., Nakayama, G., Sugimoto, H., Nomoto, S., Fujiwara, M., & Kodera, Y. (2016). Short-term outcomes after conventional transthoracic esophagectomy. *Nagoya J Med Sci*, *78*(1), 69-78.

Oh, H. K., Ihn, M. H., Son, I. T., Park, J. T., Lee, J., Kim, D. W., & Kang, S. B. (2016). Factors associated with failure of enhanced recovery programs after laparoscopic colon cancer surgery: a single-center retrospective study. *Surg Endosc*, *30*(3), 1086-1093. https://doi.org/10.1007/s00464-015-4302-y

Pederson, J. L., Padwal, R. S., Warkentin, L. M., Holroyd-Leduc, J. M., Wagg, A., & Khadaroo, R. G. (2020). The impact of delayed mobilization on post-discharge outcomes after emergency abdominal surgery: A prospective cohort study in older patients. *PLoS One*, *15*(11), e0241554. https://doi.org/10.1371/journal.pone.0241554

Porserud, A., Aly, M., Nygren-Bonnier, M., & Hagströmer, M. (2023). Association between early mobilisation after abdominal cancer surgery and postoperative complications. *Eur J Surg Oncol*, *49*(9), 106943. https://doi.org/10.1016/j.ejso.2023.05.018

Prabhakaran, S., Misra, S., Magila, M., Kumar, S. S., Kasthuri, S., Palanivelu, C., & Raj, P. P. (2020). Randomized Controlled Trial Comparing the Outcomes of Enhanced Recovery After Surgery and Standard Recovery Pathways in Laparoscopic Sleeve Gastrectomy. *Obes Surg*, *30*(9), 3273-3279. https://doi.org/10.1007/s11695-020-04585-2

Qin, P. P., Jin, J. Y., Min, S., Wang, W. J., & Shen, Y. W. (2022). Association Between Health Literacy and Enhanced Recovery After Surgery Protocol Adherence and Postoperative Outcomes Among Patients Undergoing Colorectal Cancer Surgery: A Prospective Cohort Study. *Anesth Analg*, *134*(2), 330-340. https://doi.org/10.1213/ane.0000000000005829

Quinn, L. M., Mann, K., Jones, R. P., Bathla, S., Stremitzer, S., Dunne, D. F., Lacasia, C., Fenwick, S. W., & Malik, H. Z. (2019). Defining enhanced recovery after resection of peri-hilar cholangiocarcinoma. *Eur J Surg Oncol*, *45*(8), 1439-1445. https://doi.org/10.1016/j.ejso.2019.03.033

Raue, W., Haase, O., Junghans, T., Scharfenberg, M., Müller, J. M., & Schwenk, W. (2004). 'Fast-track' multimodal rehabilitation program improves outcome after laparoscopic sigmoidectomy: a controlled prospective evaluation. *Surg Endosc*, *18*(10), 1463-1468. https://doi.org/10.1007/s00464-003-9238-y

Richardson, J., Di Fabio, F., Clarke, H., Bajalan, M., Davids, J., & Abu Hilal, M. (2015). Implementation of enhanced recovery programme for laparoscopic distal pancreatectomy: feasibility, safety and cost analysis. *Pancreatology*, *15*(2), 185-190. https://doi.org/10.1016/j.pan.2015.01.002

Rosowicz, A., Brody, J. S., Lazar, D. J., Bangla, V. G., Panahi, A., Nobel, T. B., Dexter-Meldrum, J., & Divino, C. M. (2023). Early Ambulation is Associated with Improved Outcomes Following Colorectal Surgery. *Am Surg*, *89*(12), 5225-5233. https://doi.org/10.1177/00031348221142590

Roulin, D., Melloul, E., Wellg, B. E., Izbicki, J., Vrochides, D., Adham, M., Hübner, M., & Demartines, N. (2020). Feasibility of an Enhanced Recovery Protocol for Elective Pancreatoduodenectomy: A Multicenter International Cohort Study. *World J Surg*, *44*(8), 2761-2769. https://doi.org/10.1007/s00268-020-05499-x

Roulin, D., Muradbegovic, M., Addor, V., Blanc, C., Demartines, N., & Hübner, M. (2017). Enhanced Recovery after Elective Colorectal Surgery - Reasons for Non-Compliance with the Protocol. *Dig Surg*, *34*(3), 220-226. https://doi.org/10.1159/000450685

Roulin, D., Blanc, C., Muradbegovic, M., Hahnloser, D., Demartines, N., & Hübner, M. (2014). Enhanced recovery pathway for urgent colectomy. *World J Surg*, *38*(8), 2153-2159. https://doi.org/10.1007/s00268-014-2518-y

Scharfenberg, M., Raue, W., Junghans, T., & Schwenk, W. (2007). "Fast-track" rehabilitation after colonic surgery in elderly patients--is it feasible? *Int J Colorectal Dis*, *22*(12), 1469-1474. https://doi.org/10.1007/s00384-007-0317-8

Schrempf, M. C., Zanker, J., Arndt, T. T., Vlasenko, D., Anthuber, M., Müller, G., Sommer, F., & Wolf, S. (2023). Immersive Virtual Reality Fitness Games to Improve Recovery After Colorectal Surgery: A Randomized Single Blind Controlled Pilot Trial. *Games Health J*, *12*(6), 450-458. https://doi.org/10.1089/g4h.2023.0004

Schuring, N., Geelen, S. J. G., van Berge Henegouwen, M. I., Steenhuizen, S. C. M., van der Schaaf, M., van der Leeden, M., & Gisbertz, S. S. (2023). Early mobilization after esophageal cancer surgery: a retrospective cohort study. *Dis Esophagus*, *36*(6). https://doi.org/10.1093/dote/doac085

Schwab, M., Brindl, N., Studier-Fischer, A., Tu, T., Gsenger, J., Pilgrim, M., Friedrich, M., Frey, P. E., Achilles, C., Leuck, A., Bürgel, T., Feisst, M., Klose, C., Tenckhoff, S., Dörr-Harim, C., & Mihaljevic, A. L. (2020). Postoperative complications and mobilisation following major abdominal surgery with vs. without fitness tracker-based feedback (EXPELLIARMUS): study protocol for a student-led multicentre randomised controlled trial (CHIR-Net SIGMA study group). *Trials*, *21*(1), 293. https://doi.org/10.1186/s13063-020-4220-8

Schwenk, W., Günther, N., Wendling, P., Schmid, M., Probst, W., Kipfmüller, K., Rumstadt, B., Walz, M. K., Engemann, R., & Junghans, T. (2008). "Fast-track" rehabilitation for elective colonic surgery in Germany--prospective observational data from a multi-centre quality assurance programme. *Int J Colorectal Dis*, *23*(1), 93-99. https://doi.org/10.1007/s00384-007-0374-z

Shen, Y., Lv, F., Min, S., Wu, G., Jin, J., Gong, Y., Yu, J., Qin, P., & Zhang, Y. (2021). Impact of enhanced recovery after surgery protocol compliance on patients' outcome in benign hysterectomy and establishment of a predictive nomogram model. *BMC Anesthesiol*, *21*(1), 289. https://doi.org/10.1186/s12871-021-01509-0

Sheth, K., Patel, S., Sheikh, A., & Pereira, B. (2024). COMPARATIVE STUDY ON INTRA-OPERATIVE AND POSTOPERATIVE OUTCOME INDICATORS BY VESSEL SEALERS VS TOTAL LAPAROSCOPIC HYSTERECTOMY [Article]. *Journal of Cardiovascular Disease Research*, *15*(4), 691-707. https://doi.org/10.48047/jcdr.2024.15.04.73

Shetiwy, M., Fady, T., Shahatto, F., & Setit, A. (2017). Standardizing the protocols for enhanced recovery from colorectal cancer surgery: Are we a step closer to ideal recovery? [Article]. *Annals of Coloproctology*, *33*(3), 86-92. https://doi.org/10.3393/ac.2017.33.3.86

Silva, Y. R., Li, S. K., & Rickard, M. J. (2013). Does the addition of deep breathing exercises to physiotherapy-directed early mobilisation alter patient outcomes following high-risk open upper abdominal surgery? Cluster randomised controlled trial [Randomized Controlled Trial]. *Physiotherapy (London)*, *99*(3), 187-193. https://sll.idm.oclc.org/login?url=https://search.ebscohost.com/login.aspx?direct=true&db=amed&AN=0172043&lang=sv&site=ehost-live

Simsek Yaban, Z., Bulbuloglu, S., Kapikiran, G., Gunes, H., Kula Sahin, S., & Saritas, S. (2024). The effect of bed exercises following major abdominal surgery on early ambulation, mobilization, pain and anxiety: A randomized‐controlled trial. *International Wound Journal*, *21*(2), 1-10. https://doi.org/10.1111/iwj.14406

Sinha, R., Verma, N., Bana, R., Kalidindi, N., Sampurna, S., & Mohanty, G. (2023). Intra-and post-operative outcomes in benign gynaecologic surgeries before and after the implementation of enhanced recovery after surgery protocols: A comparison [Article]. *Journal of Minimal Access Surgery*, *19*(1), 112-119. https://doi.org/10.4103/jmas.jmas_42_22

Song, J. X., Tu, X. H., Wang, B., Lin, C., Zhang, Z. Z., Lin, L. Y., & Wang, L. (2014). "Fast track" rehabilitation after gastric cancer resection: experience with 80 consecutive cases. *BMC Gastroenterol*, *14*, 147. https://doi.org/10.1186/1471-230x-14-147

Souza Possa, S., Braga Amador, C., Meira Costa, A., Takahama Sakamoto, E., Seiko Kondo, C., Maida Vasconcellos, A. L., Moran de Brito, C. M., & Pereira Yamaguti, W. (2014). Implementation of a guideline for physical therapy in the postoperative period of upper abdominal surgery reduces the incidence of atelectasis and length of hospital stay. *Rev Port Pneumol*, *20*(2), 69-77. https://doi.org/10.1016/j.rppneu.2013.07.005

Stone, A. B., Grant, M. C., Lau, B. D., Hobson, D. B., Streiff, M. B., Haut, E. R., Wu, C. L., & Wick, E. C. (2017). Thoracic Epidural Anesthesia and Prophylactic Three Times Daily Unfractionated Heparin Within an Enhanced Recovery After Surgery Pathway for Colorectal Surgery. *Reg Anesth Pain Med*, *42*(2), 197-203. https://doi.org/10.1097/aap.0000000000000542

Svensson-Raskh, A., Schandl, A. R., Ståhle, A., Nygren-Bonnier, M., & Olsén, M. F. (2021). Mobilization Started Within 2 Hours After Abdominal Surgery Improves Peripheral and Arterial Oxygenation: A Single-Center Randomized Controlled Trial. *PTJ: Physical Therapy & Rehabilitation Journal*, *101*(5), 1-11. https://doi.org/10.1093/ptj/pzab094

T, M. L., Rekha, K., J, S. k., Kabilan, R., Preethi, G., & Yogeshwaran, L. (2024). The Effects of Early Mobilization Protocol on Pre and Post Operative Functional Performance for Individuals with Post Abdominal Surgery. *Indian Journal of Physiotherapy & Occupational Therapy*, *18*, 183-188. https://doi.org/10.37506/shbbpp68

Takamoto, T., Hashimoto, T., Inoue, K., Nagashima, D., Maruyama, Y., Mitsuka, Y., Aramaki, O., & Makuuchi, M. (2014). Applicability of enhanced recovery program for advanced liver surgery. *World J Surg*, *38*(10), 2676-2682. https://doi.org/10.1007/s00268-014-2613-0

Tang, J. H., Wang, B., Chow, J. L. J., Joseph, P. M., Chan, J. Y., Abdul Rahman, N., Low, Y. H., Tan, Y. P., & Shelat, V. G. (2021). Improving postoperative mobilisation rates in patients undergoing elective major hepatopancreatobiliary surgery. *Postgrad Med J*, *97*(1146), 239-247. https://doi.org/10.1136/postgradmedj-2020-138650

Tankel, J., Kammili, A., Meng, A., Dehghani, M., Sakalla, R., Spicer, J., Najmeh, S., Cools-Lartigue, J., Ferri, L., & Mueller, C. (2024). Enhanced recovery after surgery after radical gastrectomy: Improved compliance over time is associated with a shorter postoperative hospital stay. *World J Surg*, *48*(2), 261-270. https://doi.org/10.1002/wjs.12055

Teeuwen, P. H., Bleichrodt, R. P., de Jong, P. J., van Goor, H., & Bremers, A. J. (2011). Enhanced recovery after surgery versus conventional perioperative care in rectal surgery. *Dis Colon Rectum*, *54*(7), 833-839. https://doi.org/10.1007/DCR.0b013e318216067d

Teeuwen, P. H., Bleichrodt, R. P., Strik, C., Groenewoud, J. J., Brinkert, W., van Laarhoven, C. J., van Goor, H., & Bremers, A. J. (2010). Enhanced recovery after surgery (ERAS) versus conventional postoperative care in colorectal surgery. *J Gastrointest Surg*, *14*(1), 88-95. https://doi.org/10.1007/s11605-009-1037-x

Thompson, E. G., Gower, S. T., Beilby, D. S., Wallace, S., Tomlinson, S., Guest, G. D., Cade, R., Serpell, J. S., & Myles, P. S. (2012). Enhanced recovery after surgery program for elective abdominal surgery at three Victorian hospitals. *Anaesth Intensive Care*, *40*(3), 450-459. https://doi.org/10.1177/0310057x1204000310

Thörn, R. W., Stepniewski, J., Hjelmqvist, H., Forsberg, A., Ahlstrand, R., & Ljungqvist, O. (2022). Supervised Immediate Postoperative Mobilization After Elective Colorectal Surgery: A Feasibility Study. *World J Surg*, *46*(1), 34-42. https://doi.org/10.1007/s00268-021-06347-2

Trowbridge, E. R., Evans, S. L., Sarosiek, B. M., Modesitt, S. C., Redick, D. L., Tiouririne, M., Thiele, R. H., Hedrick, T. L., & Hullfish, K. L. (2019). Enhanced recovery program for minimally invasive and vaginal urogynecologic surgery. *Int Urogynecol J*, *30*(2), 313-321. https://doi.org/10.1007/s00192-018-3794-0

Underwood, T. J., Noble, F., Madhusudan, N., Sharland, D., Fraser, R., Owsley, J., Grant, M., Kelly, J. J., & Byrne, J. P. (2017). The Development, Application and Analysis of an Enhanced Recovery Programme for Major Oesophagogastric Resection. *J Gastrointest Surg*, *21*(4), 614-621. https://doi.org/10.1007/s11605-017-3363-8

van Dam, R. M., Hendry, P. O., Coolsen, M. M., Bemelmans, M. H., Lassen, K., Revhaug, A., Fearon, K. C., Garden, O. J., & Dejong, C. H. (2008). Initial experience with a multimodal enhanced recovery programme in patients undergoing liver resection. *Br J Surg*, *95*(8), 969-975. https://doi.org/10.1002/bjs.6227

Vermişli, S., Çakmak, Ö., Müezzinoğlu, T., Aslan, G., & Baydur, H. (2022). The Effect of Postoperative Early Mobilization on the Healing Process and Quality of Life Following Radical Cystectomy and Ileal Conduit: A Randomized Prospective Controlled Trial [Article]. *Journal of Urological Surgery*, *9*(1), 9-19. https://doi.org/10.4274/JUS.GALENOS.2021.2021.0065

Veziant, J., Poirot, K., Mulliez, A., Pereira, B., & Slim, K. (2020). Is an enhanced recovery program (ERP) after rectal surgery as feasible as after colonic surgery? A multicentre Francophone study of 870 rectal resections. *Langenbecks Arch Surg*, *405*(8), 1155-1162. https://doi.org/10.1007/s00423-020-02001-y

Vignali, A., Elmore, U., Guarneri, G., De Ruvo, V., Parise, P., & Rosati, R. (2021). Enhanced recovery after surgery in colon and rectal surgery: identification of predictive variables of failure in a monocentric series including 733 patients. *Updates Surg*, *73*(1), 111-121. https://doi.org/10.1007/s13304-020-00848-w

Vlug, M. S., Bartels, S. A., Wind, J., Ubbink, D. T., Hollmann, M. W., & Bemelman, W. A. (2012). Which fast track elements predict early recovery after colon cancer surgery? *Colorectal Dis*, *14*(8), 1001-1008. https://doi.org/10.1111/j.1463-1318.2011.02854.x

Wick, E. C., Galante, D. J., Hobson, D. B., Benson, A. R., Lee, K. H., Berenholtz, S. M., Efron, J. E., Pronovost, P. J., & Wu, C. L. (2015). Organizational Culture Changes Result in Improvement in Patient-Centered Outcomes: Implementation of an Integrated Recovery Pathway for Surgical Patients. *J Am Coll Surg*, *221*(3), 669-677; quiz 785-666. https://doi.org/10.1016/j.jamcollsurg.2015.05.008

Wilandika, A., Gartika, N., & Nurfarida, E. (2023). Early ambulation and dhikr complementary therapies effect on intestinal peristaltic in post-open cholecystectomy patients. *Rev Bras Enferm*, *76Suppl 4*(Suppl 4), e20220636. https://doi.org/10.1590/0034-7167-2022-0636

Wilnerzon Thörn, R. M., Forsberg, A., Stepniewski, J., Hjelmqvist, H., Magnuson, A., Ahlstrand, R., & Ljungqvist, O. (2024). Immediate mobilization in post-anesthesia care unit does not increase overall postoperative physical activity after elective colorectal surgery: A randomized, double-blinded controlled trial within an enhanced recovery protocol. *World J Surg*, *48*(4), 956-966. https://doi.org/10.1002/wjs.12102

Wong-Lun-Hing, E. M., van Dam, R. M., Heijnen, L. A., Busch, O. R., Terkivatan, T., van Hillegersberg, R., Slooter, G. D., Klaase, J., de Wilt, J. H., Bosscha, K., Neumann, U. P., Topal, B., Aldrighetti, L. A., & Dejong, C. H. (2014). Is current perioperative practice in hepatic surgery based on enhanced recovery after surgery (ERAS) principles? *World J Surg*, *38*(5), 1127-1140. https://doi.org/10.1007/s00268-013-2398-6

Xing, N., Wang, H., Huang, Y., & Peng, J. (2023). Enhanced recovery after surgery program alleviates neutrophil-to-lymphocyte ratio and platelet-to-lymphocyte ratio in patients undergoing gynecological surgery [Article]. *Frontiers in Medicine*, *10*. https://doi.org/10.3389/fmed.2023.1057923

Xue, X., Wang, D., Ji, Z., & Xie, Y. (2022). Enhanced recovery after surgery in patients undergoing laparoscopic partial nephrectomy. Results from a real-world randomized controlled trial [Article]. *Wideochirurgia I Inne Techniki Maloinwazyjne*, *17*(1), 116-126. https://doi.org/10.5114/wiitm.2021.108216

Yilmaz, G., Akça, A., & Aydin, N. (2018). Enhanced recovery after surgery (ERAS) versus conventional postoperative care in patients undergoing abdominal hysterectomies. *Ginekol Pol*, *89*(7), 351-356. https://doi.org/10.5603/GP.a2018.0060

Zhang, L., Wu, Q., Wang, X., Zhu, X., Shi, Y., & Wu, C. J. (2024). Factors impacting early mobilization according to the Enhanced Recovery After Surgery guideline following gastrointestinal surgery: A prospective study. *Geriatr Gerontol Int*, *24*(2), 234-239. https://doi.org/10.1111/ggi.14799

Zhang, Y., Gong, Z., & Chen, S. (2021). Clinical application of enhanced recovery after surgery in the treatment of choledocholithiasis by ERCP. *Medicine (Baltimore)*, *100*(8), e24730. https://doi.org/10.1097/md.0000000000024730

Zhao, J., Hu, J., Jiang, Z., Wang, G., Liu, J., Wang, H., Fang, P., Liu, X., Wang, J., & Li, J. (2018). Outcome of Discharge Within 72 Hours of Robotic Gastrectomy Using Enhanced Recovery After Surgery Programs. *J Laparoendosc Adv Surg Tech A*, *28*(11), 1279-1286. https://doi.org/10.1089/lap.2018.0051

Zhu, Q., Yang, J., Zhang, Y., Ni, X., & Wang, P. (2021). Early mobilization intervention for patient rehabilitation after renal transplantation [Article]. *American Journal of Translational Research*, *13*(6), 7300-7305. https://www.embase.com/search/results?subaction=viewrecord&id=L2013389239&from=export
