# Supplementary material for: Early mobilisation after abdominal surgery: a concept analysis
Source: BMJ Open. 2026 Apr 17;16(4):e107830. doi: 10.1136/bmjopen-2025-107830 (PMC13110540; doi:10.1136/bmjopen-2025-107830)
Supplement: Supplementary file 5 [file bmjopen-2025-107830.reviewer_comments.pdf]

PEER REVIEW HISTORY

BMJ Open publishes all reviews undertaken for accepted manuscripts. Reviewers are asked to complete a checklist review form and are provided with free text boxes to elaborate on their assessment. These free text comments are reproduced below.

ARTICLE DETAILS

Title (Provisional)

Early Mobilisation after Abdominal Surgery: A Concept Analysis

Authors

Schandl, Anna Regina; Siesage, Katinka; Kroksmark, Anna-Karin; Gruber – De Sousa, Ewa; Lilliecrona, Johanna; Olsen, Monika Fagevik

---

VERSION 1 - REVIEW

**Reviewer**	**1**
**Name**	**Ghane, Golnar**
**Affiliation**	**Tehran University of Medical Sciences**
**Date**	**31-Aug-2025**
**COI**	**None**

Congratulations on an interesting conceptual analysis. To improve the readers' understanding and the quality of the article, a few comments were provided:

The necessity of this study is further explained in the introduction section.

The only difference between the case model and the related one is the patient's blood pressure drop. It is better to consider the collaboration between the medical professional, the nurse, and the physiotherapist in these models.

Modify the model, related, and contrasting cases to be more relevant to the outcome.

In the introduction section, mention abdominal and cardiothoracic surgeries and present the differences from other surgeries.

The type of anesthesia the patient is important in early postoperative movement. This is not mentioned in any section. Please pay attention to the introduction, findings, and discussion sections.

An ethical statement and strategic search should be provided.

There is no discussion of factors affecting early mobilization and differences with other surgeries.

**Reviewer**	**2**
**Name**	**Khudadad, Hanan**
**Affiliation**	**Primary Health Care Corporation, Clinical Research Department**
**Date**	**15-Sep-2025**
**COI**	**None**

well-constructed and useful concept analysis that offers a clear operational definition and practical antecedents/consequences for early mobilization after abdominal and cardiothoracic surgery

**Reviewer**	**3**
**Name**	**Van de Velde, Dominique**
**Affiliation**	**Ghent University**
**Date**	**28-Jan-2026**
**COI**	**None**

While the manuscript addresses a relevant and important topic, the current study is affected by fundamental methodological limitations. The breadth and conceptual heterogeneity of the focal concept challenge the appropriateness of a traditional Walker and Avant concept analysis. Moreover, key analytical steps—particularly the identification and theoretical development of defining attributes—are insufficiently elaborated. For these reasons, I recommend major revisions. A substantial reconceptualisation of both the object of study and the methodological approach would be required before this work could be considered for publication.

The strenghts of the study:

The topic addressed is timely and highly relevant for research and practice, and the manuscript convincingly argues for its importance.

The authors demonstrate a strong ambition to bring conceptual clarity to a complex and the under-theorised area of early mobilisation.

The manuscript is generally well organised and clearly written, making the argument accessible to a broad readership.

However, there are some limitations that needs to ba acknowledged.

The central concern is that the focal concept is too broad, multidimensional, and internally heterogeneous to be adequately examined using a straightforward Walker and Avant concept analysis.

The manuscript addresses a phenomenon that appears to encompass multiple partially overlapping concepts (early mobilisation after abdominal and cardiothoracic surgery) operating at different conceptual levels (e.g., experiential, functional, contextual, and possibly normative dimensions of one concept). However, Walker and Avant's approach presupposes a relatively bounded and coherent concept (such as e.g. Mobilisation) that can be analytically decomposed into defining attributes, antecedents, and consequences. In its current form, the manuscript treats a constellation of concepts as if it were a single, unitary construct, which fundamentally undermines the suitability of the chosen method.

I just wonder whether a traditional concept analysis is the most suitable methodology for addressing a concept of this breadth. The authors do not consider alternative approaches, such as conceptual blending or theory synthesis, which may be more appropriate for analysing composite or overlapping concepts. E.g. conceptual blending,

hybrid concept development, theory synthesis, or integrative conceptual frameworks.

A second major issue is the fact that, according to Walker and Avant, the identification of defining attributes (as the core analytical step of concept analysis) is minimally developed. The manuscript provides insufficient detail on how attributes were analytically derived from the literature. The process by which data were transformed into conceptual components remains largely implicit, which undermines the methodological rigour and reproducibility of the analysis.

So therefor, a major revision is necessary.

VERSION 1 - AUTHOR RESPONSE

Reviewer: 1
Congratulations on an interesting conceptual analysis. To improve the readers' understanding and the quality of the article, a few comments were provided:

Comment 1. The necessity of this study may be further explained in the introduction section

Authors' response: We have expanded the introduction to more clearly demonstrate the necessity of the study. Specifically, we have elaborated on the gaps in current evidence regarding the timing and type of early mobilisation following abdominal surgery, as well as the clinical implications of the absence of a unified definition. The revised text reads:

Without a standard definition, decisions regarding the timing and type of mobilisation after abdominal surgery may vary between clinicians and institutions. This may lead to suboptimal recovery practices, reduced comparability of outcomes, and challenges in implementing evidence-based physiotherapy interventions (19). Developing a clear definition improves

comprehension of how early mobilisation is used, the factors influencing its implementation and its potential clinical impact.

Reference: 19. Burgess LC, Immins, T., & Wainwright, T. W. What is the role of post-operative physiotherapy in general surgical Enhanced Recovery after Surgery pathways? European Journal of Physiotherapy. 2019;21(2), 67–72

Comment 2. The only difference between the case model and the related one is the patient's blood pressure drop. It is better to consider the collaboration between the medical professional, the nurse, and the physiotherapist in these models. Modify the model, related, and contrasting cases to be more relevant to the outcome.

Authors' response: We have adopted the reviewer's suggestion and have revised the model case, the related case, and the contrary case to more clearly reflect the defining attributes of the concept and to emphasise the collaborative roles of the medical doctor, nurse, and physiotherapist.

Specifically, in the model case**,** we strengthened the description of interprofessional collaboration by detailing how the surgeon, nurse, and physiotherapist jointly assessed the patient's readiness for mobilisation and coordinated the mobilisation process. We also clarified how this teamwork contributed to the expected outcomes of early mobilisation.

The related case has been revised to better contrast with the model case by showing that although mobilisation was attempted, the patient was physiologically unstable and unable to participate actively, and despite interprofessional involvement, the defining attributes of early mobilisation were not met. This contrasts with "model" and "related" clearer and more theoretically consistent.

In the contrary case**,** we further clarified the absence of early mobilisation by highlighting organisational barriers, lack of interprofessional collaboration, and missed opportunities to mobilise the patient despite clinical stability. This revised example more clearly demonstrates a situation that is opposite to the concept.

Comment 3. In the introduction section, mention abdominal and cardiothoracic surgeries and present the differences from other surgeries

Authors' response: Firstly, in line with Reviewer 3's comment, we have removed cardiothoracic surgery from the study, as its inclusion broadened the phenomenon beyond what is appropriate for a Walker and Avant concept analysis. This change also led us to revise the introduction. In the updated text, we describe how abdominal surgery and its postoperative recovery trajectories, physiological considerations, and clinical risks differ from those associated with other surgical procedures. The following sentences have been included:

Abdominal surgery differs from many other surgical types because it elicits distinct physiological stress responses (14), carries specific postoperative risks, and requires tailored rehabilitation strategies (12). Patients may face cardiorespiratory limitations (15) and delays in mobilisation due to pain (16), incision location, impaired gastrointestinal function, or

reduced ability to generate effective ventilation and coughing (3, 17, 18) – particularly in procedures near the diaphragm.

References:14. Cusack B, Buggy DJ. Anaesthesia, analgesia, and the surgical stress response. BJA Educ. 2020;20(9):321-8. 12. Gustafsson UO, Scott MJ, Hubner M, Nygren J, Demartines N, Francis N, et al. Guidelines for Perioperative Care in Elective Colorectal Surgery: Enhanced Recovery After Surgery (ERAS(®)) Society Recommendations: 2018. World J Surg. 2019;43(3):659-95. 15. Miskovic A, Lumb AB. Postoperative pulmonary complications. Br J Anaesth. 2017;118(3):317-34. 16. Kehlet H, Dahl JB. Anaesthesia, surgery, and challenges in postoperative recovery. Lancet. 2003;362(9399):1921-8. 3.Kehlet H, Wilmore DW. Fast-track surgery. Br J Surg. 2005;92(1):3-4. 17. Hedenstierna G, Edmark L. The effects of anesthesia and muscle paralysis on the respiratory system. Intensive Care Med. 2005;31(10):1327-35. 18. Smetana GW, Lawrence VA, Cornell JE. Preoperative pulmonary risk stratification for noncardiothoracic surgery: systematic review for the American College of Physicians. Ann Intern Med. 2006;144(8):581-95.

Comment 4. The type of anesthesia the patient is important in early postoperative movement. This is not mentioned in any section. Please pay attention to the introduction, findings, and discussion sections.

Authors' response: We agree that several additional factors may influence a patient's ability to mobilise after surgery. To address this, we have expanded both the Introduction and Discussion. In the revised Introduction, we now explicitly acknowledge how variations in anaesthesia methods, surgical techniques, patient characteristics, and clinical contexts complicate the absence of a universal definition of early mobilisation.

In the Discussion, we have added a new paragraph elaborating on the influence of different anaesthesia methods on early postoperative mobilisation, noting that anaesthetic techniques can differentially affect pain levels, respiratory function, motor block, and the patient's overall readiness to participate in mobilisation.

Comment 5. An ethical statement and strategic search should be provided.

Authors' response: We have added an ethical statement to the manuscript (see below). Additional details regarding the strategic search process are provided in Figure 1, which outlines the selection of included articles, and in Supplementary File 1, which presents the full search strings and corresponding results.

Ethics statements: This study did not involve human participants. As it is a concept analysis, no interventions, observations, or recruitment procedures were conducted. In accordance with standard requirements for conceptual and theoretical research, approval from an Institutional Ethical Review Board was therefore not required.

Comment 6. There is no discussion of factors affecting early mobilization and differences with other surgeries

Authors' response: We agree that the original manuscript did not sufficiently address factors influencing early mobilisation or variations across surgical procedures. In response, we have expanded the discussion section to include a paragraph outlining key patient-, procedure-, and system-related factors that influence the timing and feasibility of early mobilisation. The new paragraph reads:

Important patient -related considerations include pre-existing comorbidities (e.g., cardiopulmonary disease, frailty), postoperative physiological stability, effectiveness of pain management, and the patient´s level of alertness (44). Anesthesia techniques also play a significant role, as different methods can variably impact pain levels, respiratory function, motor block, and overall readiness to engage in mobilisation (45). Beyond patient characteristics, variability in surgical procedures substantially affects the timing of mobilization. For example, early mobilisation is often more achievable after minimally invasive abdominal procedures that after major surgeries that may involve greater postoperative respiratory compromise, closer hemodynamic monitoring, or the presence of drains and lines that restrict movement (46, 47). In addition, the attitudes, knowledge and clinical practices of healthcare professionals influence how consistently early mobilization is implemented (39). Take together, these differences across patient, surgical and organisational contexts underscore the challenge of establishing a unified definition of early mobilisation.

References: 44. Canet J, Gallart L, Gomar C, Paluzie G, Vallès J, Castillo J, et al. Prediction of postoperative pulmonary complications in a population-based surgical cohort. Anesthesiology. 2010;113(6):1338-50.
45. Cashman JN, Dolin SJ. Respiratory and haemodynamic effects of acute postoperative pain management: evidence from published data. Br J Anaesth. 2004;93(2):212-23.
46. Willner A, Teske C, Hackert T, Welsch T. Effects of early postoperative mobilization following gastrointestinal surgery: systematic review and meta-analysis. BJS Open. 2023;7(5). 47. Kokotovic D, Berkfors A, Gögenur I, Ekeloef S, Burcharth J. The effect of postoperative respiratory and mobilization interventions on postoperative complications following abdominal surgery: a systematic review and meta-analysis. Eur J Trauma Emerg Surg. 2021;47(4):975-90. 39. Svensson-Raskh A, Fagevik Olsén M, Nygren-Bonnier M, Schandl A. Healthcare professionals' experiences of mobilising adult patients out of bed shortly after major abdominal surgery – a qualitative study. European Journal of Physiotherapy. 2023:1-7.

Reviewer: 2

Comments 1: well-constructed and useful concept analysis that offers a clear operational definition and practical antecedents/consequences for early mobilization after abdominal and cardiothoracic surgery

Authors' reply: Thank you for your positive and encouraging feedback. We appreciate your recognition of the usefulness of our concept analysis. Your supportive comments reinforce the value of our work and its potential contribution to advancing early mobilisation practices in abdominal surgery.

Reviewer: 3
While the manuscript addresses a relevant and important topic, the current study is affected by fundamental methodological limitations.

The breadth and conceptual heterogeneity of the focal concept challenge the appropriateness of a traditional Walker and Avant concept analysis. Moreover, key analytical steps—particularly the identification and theoretical development of defining attributes—are insufficiently elaborated. For these reasons, I recommend major revisions.

A substantial reconceptualisation of both the object of study and the methodological approach would be required before this work could be considered for publication. The strengths of the study: The topic addressed is timely and highly relevant for research and practice, and the manuscript convincingly argues for its importance. The authors demonstrate a strong ambition to bring conceptual clarity to a complex and under-theorised area of early mobilisation. The manuscript is generally well organised and clearly written, making the argument accessible to a broad readership.
However, there are some limitations that need to be acknowledged.

Comment 1. The central concern is that the focal concept is too broad, multidimensional, and internally heterogeneous to be adequately examined using a straightforward Walker and Avant concept analysis. The manuscript addresses a phenomenon that appears to encompass multiple partially overlapping concepts (early mobilisation after abdominal and cardiothoracic surgery) operating at different conceptual levels (e.g., experiential, functional, contextual, and possibly normative dimensions of one concept). However, Walker and Avant's approach presupposes a relatively bounded and coherent concept (such as e.g. Mobilisation) that can be analytically decomposed into defining attributes, antecedents, and consequences. In its current form, the manuscript treats a constellation of concepts as if it were a single, unitary construct, which fundamentally undermines the suitability of the chosen method.

I just wonder whether a traditional concept analysis is the most suitable methodology for addressing a concept of this breadth. The authors do not consider alternative approaches, such as conceptual blending or theory synthesis, which may be more appropriate for analysing composite or overlapping concepts. E.g. conceptual blending,
hybrid concept development, theory synthesis, or integrative conceptual frameworks

Authors' response: We agree that combining abdominal and cardiothoracic surgery introduced unnecessary heterogeneity, which broadened the phenomenon beyond what is optimal for a Walker and Avant concept analysis. In response to this feedback, we have narrowed the focus of the manuscript to early mobilisation after abdominal surgery only**.** This revision ensures that the concept under analysis is both conceptually coherent and sufficiently bounded for the chosen methodological approach.

To further strengthen the methodological alignment, we have clarified the conceptual boundaries of the concept. Early mobilisation is now explicitly defined as a single clinical practice concept centred on the initiation of active physical movement after abdominal surgery. Elements such as haemodynamic stability, pain control, staffing, and organisational conditions are now explicitly categorised as antecedents or contextual prerequisites**,** rather than as components of separate or overlapping concepts. We hope this addresses the concern that the original version appeared to treat a constellation of concepts as a single construct.

We have also added a justification for selecting Walker and Avant's concept analysis in the Methods section, explaining its relevance for clarifying clinical practice concepts that lack definitional consistency. In addition, the Discussion now includes a methodological reflection acknowledging alternative frameworks and explaining why these approaches were not selected.

Comment 2. A second major issue is the fact that, according to Walker and Avant, the identification of defining attributes (as the core analytical step of concept analysis) is minimally developed. The manuscript provides insufficient detail on how attributes were analytically derived from the literature. The process by which data were transformed into conceptual components remains largely implicit, which undermines the methodological rigour and reproducibility of the analysis.

Authors' response: We agree that the analytical process was not sufficiently explicit in the original submission. To address this, we have made two substantial revisions:

1. We have added a detailed description in the method section of how data from the included literature was analysed. This new section explains the stepwise process of data extraction, comparison, categorisation, and synthesis that we used to derive preliminary conceptual categories. It also clarifies the criteria used to identify characteristics as defining attributes.
2. We have revised the presentation of the defining attributes to more clearly explain how they were analytically derived from the literature. This includes clarifying how repeated patterns, frequency of concepts across studies, semantic similarities, and alignment with theoretical descriptions informed the final selection of attributes.

VERSION 2 - REVIEW

**Reviewer**	**3**
**Name**	**Van de Velde, Dominique**
**Affiliation**	**Ghent University**
**Date**	**16-Mar-2026**
**COI**	

The authors did a good job to review their paper. This is now sufficient to proceed towards publication.
